# Ethylmethylhydroxypyridine Succinate Is an Inhibitor but Not a Substrate of ABCB1 and SLCO1B1

**DOI:** 10.3390/ph16111529

**Published:** 2023-10-27

**Authors:** Aleksey V. Shchulkin, Pelageya D. Erokhina, Anna V. Goncharenko, Pavel Yu. Mylnikov, Ivan V. Chernykh, Yulia V. Abalenikhina, Maria S. Kotliarova, Elena N. Yakusheva

**Affiliations:** 1 Department of Pharmacology, Ryazan State Medical University, 390026 Ryazan, Russia; 2 Bach Institute of Biochemistry, Federal Research Centre “Fundamentals of Biotechnology”, Russian Academy of Sciences, 119071 Moscow, Russia; pylaevanna@gmail.com (A.V.G.);

**Keywords:** Ethylmethylhydroxypyridine succinate, ABCB1, SLCO1B1, drug–drug interaction

## Abstract

2-Ethyl-6-methyl-3-hydroxypyridine succinate (EMHPS, Mexidol) is an original antioxidant and an anti-ischemic drug with the possibility of wide applications in the complex therapy of diseases, accompanied by the development of oxidative stress and ischemia; for example, ischemic stroke, chronic cerebral ischemia, and chronic heart failure. The use of EMHPS in the complex therapy of the above diseases may cause the development of drug–drug interactions, particularly pharmacokinetic interactions at the level of transporter proteins. In the present study, we evaluated the interaction of EMHPS with ABCB1 and SLCO1B1. In Caco-2 cells, it was shown that EMHPS is not a substrate of ABCB1 and that it does not affect its expression, but at the same time, it inhibits the activity of this transporter. Its inhibitory activity was inferior to verapamil—a classic inhibitor of ABCB1. In HEK293 and HEK293-SLCO1B1 cells, it was shown that EMHPS is not a substrate of SLCO1B1 either, but that it inhibited the activity of the transporter. However, its inhibitory activity was inferior to the classic inhibitor of SLCO1B1-rifampicin. Furthermore, it was found out that EMHPS does not affect SLCO1B1 expression in HepG2 cells. The approach proposed by the FDA (2020) and the International Transporter Consortium (2010) was used to assess the clinical significance of the study results. The effect of EMHPS on SLCO1B1 and the systemic inhibition of ABCB1 by EMPHS are not clinically significant, but ABCB1 inhibition by EMHPS in the gastrointestinal tract should be tested in vivo through clinical trials.

## 1. Introduction

**Figure 1:**
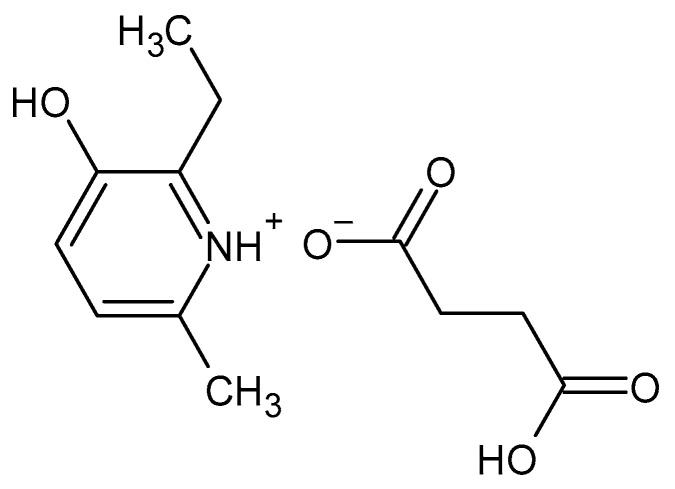
Structure of 2-ethyl-6-methyl-3-hydroxypyridine succinate.

2-Ethyl-6-methyl-3-hydroxypyridine succinate (EMHPS, Mexidol) is an original antioxidant and anti-ischemic drug with pronounced neuroprotective activity (Figure 1) [1,2].

The antioxidant properties of EMHPS are caused by hydroxypyridine and are determined via its radical scavenging activity and the suppression of lipid peroxidation [3,4,5]. The succinate in the molecule of EMHPS is an energy-rich substrate, undergoing oxidation via succinate dehydrogenase in mitochondrial enzyme complex II. During ischemia, this oxidative process counteracts the reduction of mitochondrial enzyme complex I, thereby maintaining the synthesis of ATP [6]. Moreover, succinate can activate its specific receptors (SUCNR1) and initiate different metabolic cascades that increase tissue resistance to hypoxia [7].

EMHPS is used for the treatment of acute disorders of cerebral circulation, craniocerebral trauma, encephalopathy, and a number of other diseases. In a randomized double blind multicenter placebo-controlled, parallel groups clinical trial (EPICA) on 151 patients with hemispheric ischemic stroke, it was shown that EMHPS administration improved the neurological status of patients compared with the placebo [8]. The statistically significant benefit of EMHPS over the placebo was confirmed through basic neurological tests in a multicenter, randomized double-blind trial including 318 patients with chronic brain ischemia [9]. A number of studies have also shown the possibility of using EMHPS in the complex therapy of chronic heart failure [10].

The use of EMHPS in the complex therapy of the above diseases may cause the development of drug–drug interactions (DDIs), particularly pharmacokinetic interactions. During the development of the drug, it was shown that ethylmethylhydroxypyridine succinate is an inducer of the CYP3A4 isoenzyme [11], and the main route of its metabolism is the formation of glucuronoconjugate [12]. Therefore, the logical step in further research is to study its affiliation with the substrate/inhibitors/inducers of transporters.

Drugs are transported by two superfamilies of transporters: single solute carrier (SLC) transporters and ATP-binding cassette (ABC) transporters. As they play crucial roles in absorption, distribution, metabolism, and elimination (ADME), these drug transporters are of great pharmacological importance. Transporters take part in the transmembrane translocation of drugs in the intestine, liver, kidneys, and other organs [13]. Drugs can potentially compete with each other for binding the transporters, which can lead to unexpected changes in serum and tissue drug levels, and cause possible toxic side-effects.

In the current study, we evaluated the interaction of EMHPS with ABCB1 and SLCO1B1. ABCB1 (also known as P-glycoprotein, MDR1) is a transmembrane protein belonging to the ATP-binding cassette transporter (ABC transporter) family. ABCB1 is an efflux pump that uses the energy of ATP and transports a large number of compounds (structurally diverse) out of the cells [14,15].

ABCB1 is localized on the biliary membrane of hepatocytes, where it mediates the excretion of substrates into bile; on the luminal membrane of enterocytes, where it limits the absorption of drugs from the intestine; and on the luminal membrane of proximal renal tubule cells, where it promotes the renal secretion of drugs [16]. ABCB1 reduces the permeability of many pharmaceutical compounds across the blood–brain barrier into the brain [17]. Thus, ABCB1 inhibitors are thought to enhance substrate absorption and to reduce their elimination [18].

The organic anion-transporting polypeptide SLCO1B1, also known as LST1, SLCO1B1, OATP2, OATPC, SLC21A6, is a part of the SLC superfamily and is highly expressed on the hepatocyte basolateral membrane. SLCO1B1 mediates the sodium-independent uptake of drugs at the sinusoidal pole of hepatocytes [19]. Several studies have shown that SLCO1B1 is involved in DDI that leads to change the systemic exposure of its substrates [20].

Both ABCB1 and SLCO1B1 can be involved in DDI. Thus, a potential role of new molecules as ABCB1 and SLCO1B1 substrates or inhibitors is recommended for evaluation in vitro [21,22].

The effect of EMHPS on the activity and expression of ABCB1 in the gut has not been properly studied yet. Also, it is unknown as to whether EMHPS is a substrate of ABCB1.

EMHPS is extensively metabolized in the liver to 2-ethyl-6-methyl-3-hydroxypyridine glucuronide and 2-ethyl-6-methyl-3-hydroxypyridine phosphate, which have no pharmacological activity [12]. Since the liver plays an important role in the elimination of EMHPS, it is advisable to test whether this drug is an SLCO1B1 substrate or not. Studies testing the EMHPS effect on SLCO1B1 activity have not been conducted either.

The aim of the work is to research EMHPS properties as a potential ABCB1 and SLCO1B1 inhibitor, and to further characterize EMHPS as a potential factor for altered drug disposition in humans. Also, in the study, we tested the affiliation of EMHPS to the substrates of these transporters. We used cell lines expressing the human transporters ABCB1, SLCO1B1 and fexofenadine, and atorvastatin as prototypical substrates of the respective transporters.

## 2. Results

### 2.1. MTT Assay

EMHPS at all tested concentrations of 1–500 µM had no effect on the viability of HEK293 and Caco-2 cells. This parameter ranged from 85 to 105% compared to the control. The obtained results indicate that the test substance is non-toxic.

### 2.2. EMHPS Transport through the Caco-2 Cells Monolayer

To test the participation of ABCB1 in the transport of EMHPS, EMHPS transport through the Caco-2 cells monolayer was evaluated. Papp b-a (characterizing the basal-to-apical transport) and Papp a-b (characterizing apical-to-basal transport) of EMHPS, and the efflux ratio (ER) of Papp b-a/Papp a-b were investigated. The ER was used to test the permeability through the Caco-2 cells monolayer. An ER of higher than 2.0 means an increase in the transport of the analyzed compound from the basolateral to the apical chamber with the involvement of efflux transporters (for example, ABCB1 or the breast cancer resistance protein—ABCG2), while an ER between 0.5 and 2.0 means that there is no directed transport. There was no difference between transepithelial transport of EMHPS at all concentrations (10, 100, and 250 µM) from the basal-to-apical chamber, and from the apical-to-basal chamber. The ER was around 1 (Table 1). These results indicate that EMHPS is not a substrate of ABCB1.

### 2.3. EMHPS Transport in HEK293-SLCO1B1 Cells

**Figure 2:**
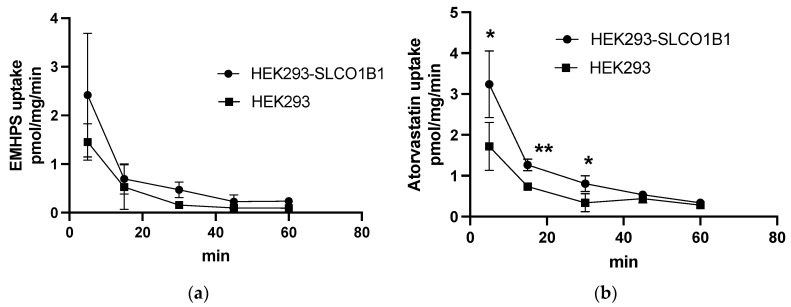
EMHPS 10 µM (**a**) and atorvastatin 1 µM (**b**) uptake in HEK293-SLCO1B1 and HEK293 cells. There was no difference in EMHPS uptake in HEK293-SLCO1B1 and HEK293, but 1 µM atorvastatin uptake was higher in HEK293-SLCO1B1. * *p* < 0.05, ** *p* < 0.01—statistically significant differences between atorvastatin uptake in HEK293-SLCO1B1 and HEK293, Student’s *t*-test.

To test the participation of SLCO1B1 in the transport of EMHPS, EMHPS uptake in HEK293-SLCO1B1 and HEK293 was evaluated. There was no difference in the accumulation of 10 µM EMHPS in HEK293-SLCO1B1 and HEK293 over incubation times of 5, 15, 30, 45, and 60 min (Figure 2a). At the same time, 1 µM atorvastatin uptake was higher in HEK293-SLCO1B1 compared to HEK293 at 5, 15, and 30 min (Figure 2b). These data indicate that the HEK293-SLCO1B1 model system works properly, and that EMHPS is not a substrate of SLCO1B1 either.

### 2.4. Effect of EMHPS on the ABCB1-Mediated Transport of Fexofenadine

To examine the effect of EMHPS on ABCB1-mediated transport, the transport of the ABCB1 substrate fexofenadine through a monolayer of Caco-2 cells was tested. The transport of fexofenadine was assessed under both the absence and presence of EMHPS and the ABCB1 inhibitor, verapamil. In the absence of tested drugs, fexofenadine transport was markedly higher in the basal-to-apical direction than in the opposite direction (Table 2).

**Figure 3:**
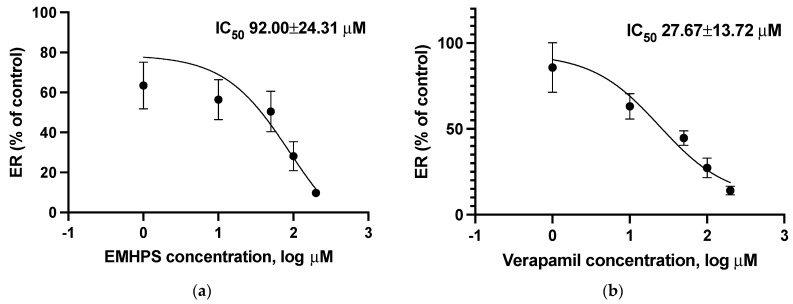
Concentration-dependent inhibition of ER (Papp b-a/Papp a-b of fexofenadine) by EMHPS (**a**) or verapamil (**b**). EMHPS and verapamil inhibit ABCB1 activity, which is characterized by changes in ER of fexofenadine.

The presence of EMHPS significantly decreased basal-to-apical fexofenadine transport at a concentration of 200 µM by 52.5% (*p* < 0.05), increased the apical-to-basal fexofenadine transport by 375.4% (*p* < 0.05), and also inhibited ER at a concentration of 50 µM by 50.2% (*p* < 0.05), 100 µM by 72.9% (*p* < 0.01), and 200 µM by 90.6% (*p* < 0.001) (Table 2). The IC_50_ value of EMHPS of ER was 92.00 ± 24.31 µM (Figure 3).

Succinic acid at all tested concentrations did not have a significant effect on all studied parameters (Papp b-a, Papp a-b, and ER) of fexofenadine transport (*p* > 0.05) (Table 3).

The addition of the ABCB1 inhibitor verapamil decreased the polarized transcellular transport of fexofenadine (decreased basal-to-apical fexofenadine transport and ER, and increased the apical-to-basal fexofenadine transport), with a verapamil IC_50_ value of ER 27.67 ± 13.72 µM (Table 4, Figure 3).

There were no significant differences between EMHPS and verapamil in their ability to inhibit ER of fexofenadine; however, the IC_50_ value of EMHPS was higher than the IC_50_ value of the classic ABCB1 inhibitor verapamil (*p* = 0.016).

Thus, EMHPS inhibits the activity of ABCB1. This effect depends on the hydroxypyridine derivative in its molecule, and its inhibitory activity is inferior to the classical ABCB1 inhibitor, verapamil.

### 2.5. Effect of EMHPS on SLCO1B1 Activity

**Figure 4:**
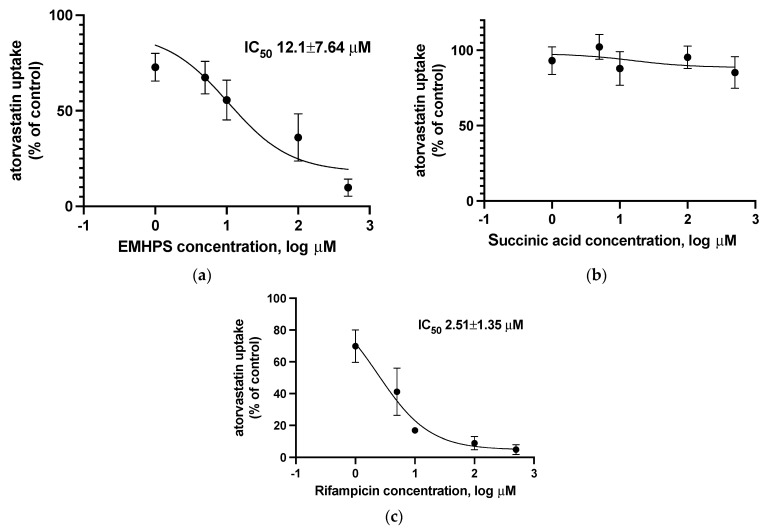
Concentration-dependent inhibition of SLCO1B1-mediated uptake of 1 µM of atorvastatin by EMHPS (**a**), succinic acid (**b**), or rifampicin (**c**).

The uptake of 1 µM atorvastatin was measured in the absence and presence of EMHPS (1–500 µM), succinic acid (1–500 µM), or rifampicin (1–500 µM) as a classic SLCO1B1 inhibitor. Atorvastatin uptake in HEK293-SLCO1B1 cells was inhibited in a concentration-dependent manner in the presence of EMHPS with IC_50_ value 12.1 ± 7.64 µM (Figure 4). However, in terms of inhibitory activity, the IC_50_ value of EMHPS was higher than the IC_50_ value of the classic OATP1B1 inhibitor rifampicin (2.51 ± 1.35 µM) (*p* = 0.049).

**Figure 5:**
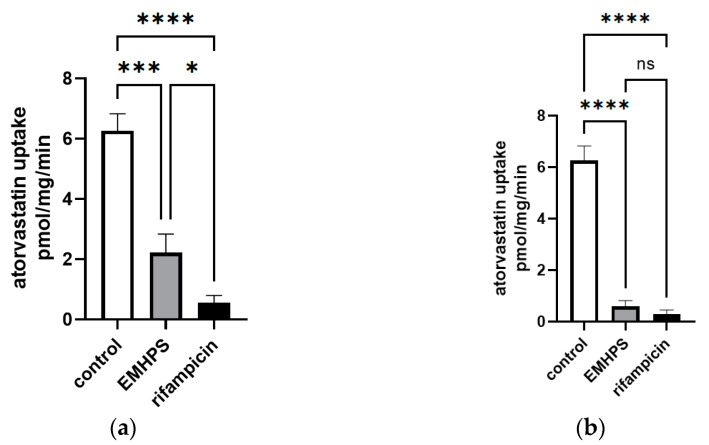
The effects of EMHPS and rifampicin, 100 µM (**a**) and 500 µM (**b**), on SLCO1B1-mediated uptake of 1 µM of atorvastatin. There was no significant difference between EMHPS and rifampicin in the ability to inhibit SLCO1B1 activity at a concentration of 500 µM. At a concentration of 100 µM, rifampicin inhibited SLCO1B1 more strongly than EMHPS. * *p* < 0.05, *** *p* < 0.001, **** *p* < 0.0001—statistically significant differences, ns—not significant, ANOVA, post hoc Tukey’s test.

EMHPS at a maximum concentration of 500 µM inhibited SLCO1B1 activity by 90.6% (*p* < 0.001), while rifampicin showed 95.4% (*p* < 0.001). The differences between the two groups were not significant (*p* > 0.05) (Figure 5). At a concentration of 100 µM, EMHPS inhibited SLCO1B1 activity by 64.7% (*p* < 0.001) and rifampicin showed 91.2% (*p* < 0.001); the differences between the drugs were significant (*p* < 0.05) (Figure 5).

Succinic acid at all tested concentrations did not have a significant effect on SLCO1B1 activity (*p* > 0.05) (Figure 4).

Thus, despite the degree of uncertainty in the IC_50_ values, EMHPS appears to be inferior to the classic inhibitor of SLCO1B1 rifampicin. The inhibitory activity of EMHPS is due to the presence of hydroxypyridine in its molecule.

### 2.6. Effect of EMHPS on Expression of ABCB1 and SLCO1B1

**Figure 6:**
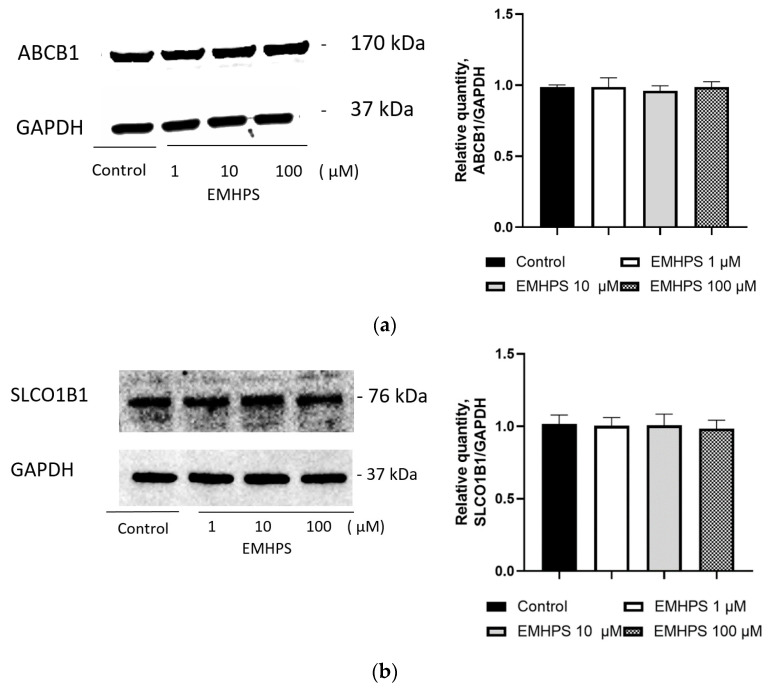
Effect of EMHPS on ABCB1 (**a**) and SLCO1B1 (**b**) expression. EMHPS at all tested concentrations (1, 10, and 100 µM) during incubation for 24 h did not significantly affect the expression of ABCB1 in Caco-2 cells and SLCO1B1 in HepG2 cells.

EMHPS could hypothetically influence ABCB1 and SLCO1B1, either by changing the expression or by changing their activity. The effect of EMHPS on protein expression was investigated in order to rule out this confounding effect. EMHPS at all tested concentrations (1, 10, and 100 µM) over 24 h incubation did not significantly affect the expression of ABCB1 in Caco-2 cells and SLCO1B1 in HepG2 cells (Figure 6).

## 3. Discussion

EMHPS is an original antioxidant and anti-ischemic drug with the possibility of wide application in the complex therapy of diseases, accompanied by the development of oxidative stress and ischemia [23]. The effectiveness of the use of EMHPS, particularly in the treatment of ischemic stroke, chronic cerebral ischemia, and chronic heart failure, was established [8,9,10]. Given the wide range of drugs used in the treatment of these diseases [24], there is a high probability of developing drug–drug interactions involving EMHPS when it is included in the therapy. Therefore, in the current study, we have tested whether EMHPS belongs to the substrates and inhibitors of the main clinically significant transport proteins, ABCB1 and SLCO1B1.

ABCB1 can recognize substrates with very different molecular weights. For example, it can transport small molecules such as cimetidine (MW 252) and small peptides such as N-acetyl-leucyl-leucyl-norleucinal (MW 383), as well as large molecules such as cyclosporine A (MW 1203) and gramicidin D (MV 1882) [25].

SLCO1B1 also transports a wide range of substrates such as statins, angiotensin II type 1 receptor antagonists, angiotensin-converting enzyme inhibitors, and several others [26].

It was shown that transport of EMHPS from the apical to the basolateral chamber was equivalent to transport from the basolateral to the apical chamber. The ER (the ratio of basal-to-apical transport to apical-to-basal transport) of EMHPS through the Caco-2 cells monolayer was around 1. These results indicate that the transport of EMHPS through the Caco-2 monolayer occurs without the participation of specific transporters, including ABCB1 [27]. Thus, EMHPS is not a substrate of ABCB1. Similarly, EMHPS is not a substrate of SLCO1B1. This is evidenced by the same EMHPS uptake in HEK293-SLCO1B1 and HEK293. At the same time, the uptake of the SLCO1B1 substrates (rosuvastatin, pitavastatin [28], or atorvastatin, in our research) is significantly higher in HEK293- SLCO1B1 cells rather than in HEK293 cells.

Our study has also determined that EMHPS inhibits the activity of ABCB1 (the IC_50_ value of ER (Papp b-a/Papp a-b) of 150 µM of fexofenadine was 92.00 ± 24.31 µM) and SLCO1B1 (the IC_50_ value of the inhibition of SLCO1B1-mediated uptake of 1 µM of atorvastatin was 12.1 ± 7.64 µM). However, succinic acid had no effect on the studied transporters; thus, the inhibitory activity of EMHPS on ABCB1 and SLCO1B1 was due to the presence of hydroxypyridine in its molecule.

The clinical significance of the study results can be assessed by the approach proposed by the FDA (2020) and The International Transporter Consortium (2010). For SLCO1B1, the ratio of Cmax of EMHPS in the blood plasma to the IC_50_ value was evaluated. If the value obtained is higher than or equal to 0.1, then this pharmacokinetic interaction between the test substances and SLCO1B1 substrates—in particular, atorvastatin—may be of clinical significance. The EMHPS Cmax is 0.39 µM [12]. The calculated value for Cmax/IC_50_ (0.39 µM/12.1 µM = 0.032) indicates that the effect of EMHPS on SLCO1B1 is most probably not clinically significant. EMHPS will not affect the activity of the studied transporters in the clinical practice; that is, they are safe drugs with regard to the development of drug–drug interactions at the level of SLCO1B1.

The obtained results are consistent with the ideas regarding the structures of clinically significant inhibitors of SLCO1B1 (IC_50_ < 10 µM). If a drug has a predicted clogP > 3.5 and ≥1 negatively charged atom, the compound is an SLCO1B1 inhibitor 94% of the time. The combination of clogP > 3.5 and ≥1 negatively charged atom provided a 25-fold enrichment of inhibitors compared to the original dataset distribution [29].

There are several approaches to translate the results of assessing the effect of test drugs on the activity of ABCB1 obtained in vitro to in vivo. Oral drugs can affect the activity of the transporter in the intestine, where the concentration is quite high, and influence ABCB1 in other organs and tissues, for which the concentration of the substance in the systemic circulation is important. Zhang et al. proposed that drugs exhibiting an [I]1/IC_50_ ≥ 0.1 or [I]2/IC_50_ ≥ 10 should be evaluated to determine whether inhibition occurs in vivo, where [I]1 is the mean drug steady-state total Cmax at the highest clinical dose, and [I]2 is the theoretical maximal gastrointestinal drug concentration after oral administration, calculated as the highest clinical dose (mg) in a volume of 250 mL [21,30].

For EMHPS, [I]1/IC_50_ was = 0.39 µM/92.00 µM = 0.0042. It means that the systemic inhibition of ABCB1 by EMPHS is most probably not clinically significant. At the same time, [I]2/IC_50_ was (0.250 g/255 g/mol/0.25 L)/92.00 µM = 42.6. It means that an inhibition of ABCB1 by EMPHS in the gastrointestinal tract is most probably clinically significant. These results indicate the possible clinical significance of ABCB1 inhibition by EMHPS and the need for clinical studies of drug–drug interactions between EMHPS and ABCB1 substrates, such as digoxin and dabigatran etexilate [31].

In the current study, it was shown that EMHPS is not an ABCB1 substrate, indicating that substrate inhibition of the activity of the transporter protein is unlikely. It can be assumed that the most likely mechanism of interaction was the allosteric inhibition of ABCB1 activity. This mechanism was previously described for drugs of the dihydropyridine series—nicardipine, nimodipine, nitrendipine, and nifedipine [32]. Currently, three substrate binding sites (transported substances) and one allosteric site, called the M site, are isolated in the ABCB1 structure; the M site changes the activity of the transporter [33]. The M site cross-reacts with both halves of the transporter protein, being able to change its conformation and thus regulate the efflux of ABCB1 substrates. More recent studies have found that allosteric inhibitors (such as tariquidar) are able to disrupt the closure of the intracellular transmembrane domain, and in general, form corresponding changes in the structure, leading to difficulty in transporting the substrate from the cell to the outside [34]. The presence of a hydrogen atom bonded to a nitrogen atom located in the aromatic cycle, two alkyl residues in the ortho-position and an oxygen-containing radical in the meta-position (during the formation of a zwitterion) can justify the similarity of the reaction sites of EMHPS and the molecules of dihydropyridine derivatives.

## 4. Materials and Methods

### 4.1. Caco-2 Cell Culture

Caco-2 is a classic model system for testing drugs for ABCB1 substrates/inhibitors, as well as for studying drug absorption [35]. The Caco-2 cell line was purchased from American Type Culture Collection (Institute of Cytology, Russian Academy of Sciences, Moscow, Russia). Caco-2 cells were cultured in Dulbecco’s modified Eagle’s medium containing 15% fetal bovine serum, 2 mM l-glutamine, 100 units/mL penicillin G, and 100 μg/mL streptomycin in an atmosphere of 5% CO_2_–95% air at 37 °C. Caco-2 cells were used to test the effect of EMHPS on the expression and activity of ABCB1, and to test EMHPS for belonging to the ABCB1 substrate.

### 4.2. HEK293-SLCO1B1 Cell Line

The SLCO1B1 gene was obtained via synthesis and was cloned into the pEGFP-N1 vector at the Xho I and Hind III sites, resulting in the pEGFP-SLCO1B1 plasmid. The proteins will be expressed as a fusion polypeptide with GFP at the C-terminus. Since the fluorescent protein will be in the same downstream reading frame as the target protein, the appearance of fluorescence will clearly indicate its expression. The HEK293 cell line was purchased from American Type Culture Collection (Institute of Cytology, Russian Academy of Sciences, Moscow, Russia). HEK293 cells were grown in Dulbecco’s modified Eagle’s medium (DMEM, Paneco) containing 10% (*v*/*v*) fetal calf serum, 2 mM glutamine, 100 units/mL penicillin G, and 100 μg/mL streptomycin at 37 °C in a humidified 5% CO_2_ atmosphere. HEK293 cells were stably transfected with the pEGFP-SLCO1B1 plasmid using Lipofectamine™ 3000 Reagent (Invitrogen, Waltham, MA, USA). To obtain stable clones expressing SLCO1B1, selection using G-418 sulfate (Promega, Singapore) at a concentration of 500 μg/mL was made. Cells were subjected to clonal selection by limiting dilution. Target clones were selected using fluorescence intensity; the synthesis of SLCO1B1 was confirmed with specific antibodies. The obtained clones were used for further work. The HEK293-SLCO1B1 cell line was used to test the effect of EMHPS on the activity of SLCO1B1, and to test for whether EMHPS belongs to the SLCO1B1 substrate.

### 4.3. HepG2 Cell Line

The HepG2 cell line was purchased from American Type Culture Collection (Institute of Cytology, Russian Academy of Sciences, Moscow, Russia). Cells were cultured in Dulbecco’s modified Eagle’s medium containing 15% fetal bovine serum, 2 mM l-glutamine, 100 units/mL penicillin G, and 100 μg/mL streptomycin in an atmosphere of 5% CO_2_ at 37 °C on a 24-well plate until a monolayer was formed. HepG2 cells were used to test the effect of EMHPS on the expression of SLCO1B1.

### 4.4. Cytotoxicity (MTT) Assay

Caco-2 and HEK93 cells were incubated in a 96-well microplate (104 cells per well) with different concentrations (1, 10, 50, 100, and 500 µM) of EMHPS for 24 h. At the end of incubation, 20 μL of 0.5% 3-(4,5-dimethylthiazol-2-yl)-2,5-diphenyltetrazolium bromide (MTT) was added to each well, followed by a 2 h incubation. The MTT solution was then aspirated and 200 μL of 1% dimethyl sulfoxide solution (PanEko) was added. Absorption was recorded after 10 min at 530 nm with a Stat Fax 2100 plate reader (Awareness Technology, Palm City, FL, USA). The survival of cells in the presence of EMHPS was calculated according to the equation: (optical density (OD) of test wells − OD of medium)/(OD of control wells − OD of medium) × 100%, where OD is the optical density [36].

### 4.5. Transport Experiments on Caco-2 Cells

Transport experiments were carried out in Transwell^®^ systems (12 mm Transwell^®^ with 0.4 µm Pore Polycarbonate Membrane Insert, Sterile, «Corning»). The Transwell^®^ system consists of two chambers, apical and basolateral. The bottom of the apical chamber (Transwell^®^ insert) is a semipermeable membrane on which Caco-2 cells were seeded at a density of about 60,000 cells/well, and grown for 21 to 23 days to reach confluence and to differentiate into enterocytes exhibiting transporter proteins and tight junctions. The medium was changed every 2–3 days. The apical and basolateral chambers represent the luminal and blood/mesenteric lymph sides of the gastrointestinal tract, respectively. Therefore, transport from the apical chamber to the basolateral is a model of substances absorption, and from the basolateral to the apical it is a model of intestinal efflux transporters work; for example, ABCB1 [37].

The transepithelial electrical resistance (TEER) across the cell monolayers was monitored using a Millicell-ERS (electrical resistance system) to assess cell monolayer integrity. For transport studies, only monolayers with a TEER > 500 Ω cm^2^ were used.

The medium in chambers was removed and the cell monolayers were washed twice in warm (37 °C) transport medium (Hanks‚ balanced salt solution containing 10 mM HEPES and 1% dimethylsulfoxide (DMSO)). The apical (for apical to basolateral transport) or basolateral (for basolateral to apical transport) chamber were filled with an equal volume of pre-warmed transport medium containing EMHPS (50, 100, and 500 μmol/L). Chamber–recipient was filled with transport medium without EMHPS. At 60, 120, and 180 min, 100 μL samples were taken from the recipient chamber and the drug concentration was analyzed. EMHPS transport was assessed in the absorptive (apical to basolateral, a-b) and secretory (b-a) directions.

The apparent permeability (Papp) of EMHPS through Caco-2 monolayers was calculated using Equation (1).

(1) $$Papp=\frac{dQ}{dt}\times \frac{1}{\left(A\times C_{0}\right)}$$

*A*, membrane surface area (1.12 cm^2^); *C*_0_, EMHPS concentration at *t* = 0 µM; and *dQ/dt,* amount of EMHPS transported within a given time period µM/s.

The ratio of Papp (b-a) and Papp (a-b), known as the efflux ratio (ER), was calculated in accordance with the following Equation (2):

(2) $$ER=\frac{Papp\;b\text{-}a}{Papp\;a\text{-}b}$$

The ER was used as a marker for permeability through Caco-2 cell monolayers.

To assess the effect of EMHPS on the activity of ABCB1, we studied the transport of fexofenadine 150 μmol/L—a transporter substrate through a monolayer of Caco-2 cells, and its change under the action of EMHPS at concentrations of 1, 10, 50, 100, and 200 µM. To study the roles of different parts of the EMHPS molecule for its action on ABCB1, experiments with succinic acid were also carried out. As a positive control, verapamil (classic inhibitor of ABCB1) was used.

EMHPS, succinic acid, or verapamil were added to both chambers (apical and basolateral), regardless of the direction of transport of fexofenadine. Prior to the transport experiments, the culture media was aspirated, and the monolayers were washed twice with transport medium and preincubated with EMHPS, succinic acid, or verapamil for 30 min.

The data are presented as the mean ± SD from three monolayers.

### 4.6. Uptake Experiments

HEK293-SLCO1B1 and HEK293 were cultured on a 24-well plate. Before the uptake experiments, cells were washed with pre-warmed (37 °C) uptake buffer containing Hanks’ Balanced Salt Solution (no phenol red) supplemented with 12.5 mM HEPES (pH 7,4) and 1% of DMSO.

EMHPS was dissolved in uptake buffer to a final concentration of 10 µM. The cells (HEK293-SLCO1B1 and HEK293) were incubated with the test solution at room temperature for 5, 15, 30, 45, and 60 min. The reaction was stopped by removing the uptake buffer containing EMHPS and adding 0.5 mL of ice-cold uptake buffer to the well. Then, the cells were immediately washed three times with 0.5 mL of ice-cold Dulbecco’s phosphate buffered saline. The cells were lysed using three “freeze-thaw” cycles (cells were frozen at −80 °C and thawed at room temperature). The uptake of atorvastatin–substrate of SLCO1B1 at a concentration of 1 µM was evaluated as a positive control.

To assess the effect of EMHPS on the activity of SLCO1B1, we studied the uptake of 1 µM atorvastatin in HEK293-SLCO1B1 and its change under the action of EMHPS at concentrations of 1, 5, 10, 50, and 100 µM. Before the transport experiment, HEK293-SLCO1B1 cells were pre-incubated with EMHPS for 15 min, followed by co-incubation with the SLCO1B1 substrate atorvastatin. To study the roles of different parts of the EMHPS molecule for its action on SLCO1B1, experiments with succinic acid were also carried out.

As a positive inhibition control, rifampicin at similar concentrations was used.

Three independent experiments were performed.

### 4.7. Fexofenadine HPLC-UV Analysis

The concentration of fexofenadine in the transport medium was determined by HPLC-UV (Styer, «Aquilon»). The resulting sample of the transport medium (50 μL) was diluted in 150 μL of the mobile phase, and 100 μL of the resulting solution was injected into the chromatograph. A Phenomenex Synergi 4u Polar-RP 80A (250 × 4.6) chromatographic column was used for analysis. The separation temperature was 35 °C; the flow rate was 1 mL/min. Composition of the mobile phase: acetonitrile (128 mL), deionized water (267.4 mL), acetic acid (6.33 mL), triethylamine to pH = 6.7. The retention time of fexofenadine under these conditions was 12.8 min. Detection was carried out at a wavelength of 220 nm [38].

### 4.8. Atorvastatin LC-MS/MS Analysis

Atorvastatin concentrations in the cell lysate were analyzed with a previously published method [39] using Ultimate 3000 Liquid Chromatograph and a TSQ Fortis triple quadrupole mass spectrometer with an electrospray ionization (ESI) source (Thermo Fisher Scientific, Rockford, IL, USA). Proteins were precipitated by adding acetonitrile in a 1:1 ratio to the sample. The chromatographic separation was performed on a C18 column UCT Selectra (C18 4.6 mm × 100 mm, 5 um, 100 A) with pre-column Selectra C18 Guard Cartridges SLC-18GDC46-5UM, using 0.1% formic acid solution as mobile phase A and acetonitrile as mobile phase B. The mobile phase gradient program was as follows: 0–0.6 min—35% A and 65% B, 0.6–5.0 min—5% A and 95% B, 5.0–8.0 min—35% A and 65% B. The flow rate and the column temperature were maintained at 300 µL/min and 35 °C. Aliquots (2 μL) of the extracted samples were injected. For quantitative analysis, the optimized selective reaction monitoring (SRM) mode with the following parameters: *m*/*z* 559.30 > 466.20, 559.30 *m*/*z* → 440.20 *m*/*z*, was used.

### 4.9. EMHPS HPLC-UV Analysis

EMHPS concentrations in the transport medium (transport experiments with Caco-2 cells) were analyzed using HPLC-UV (HPLC chromatograph Stayer) with UV detection at a wavelength of 296 nm. The sample (50 µL) was diluted in 150 µL of the mobile phase, and 100 µL of the resulting solution was injected into the chromatograph. In the analysis, a Beckman Coulter 4.6 × 150 mm, 5 μm chromatographic column was used.

The separation temperature was 35 °C. The flow rate was 0.8 mL/min. Mobile phase composition: acetonitrile and water in a 20:80 *v/v* ratio with the addition of acetic acid to pH 3.25. The retention time of EMHPS under these conditions was 5.5 min.

### 4.10. EMHPS LC-MS/MS Analysis

EMHPS concentrations in the HEK293-SLCO1B1 cells lysate were analyzed using HPLC-MS/MS (Ultimate 3000 Liquid Chromatograph and TSQ Fortis triple quadrupole mass spectrometer). Proteins were precipitated by adding methanol in a 1:1 ratio to the sample. The chromatographic separation was performed on a C18 column UCT Selectra (C18 4.6 mm × 100 mm, 3 um, 100 A) with pre-column Selectra C18 Guard Cartridges SLC-18GDC46-3UM, using formic acid solution 0.1% as mobile phase A and methanol as mobile phase B. The mobile phase gradient program was as follows: 0.0 min—90% A and 10% B, 0.01 min—70% A and 30% B, 3.5 min—50% A and 50% B, 3.5 min—10% A and 90% B, 5 min—10% A and 90% B, 5 min—90% A and 10% B, 7.5 min—90% A and 10% B. The flow rate and the column temperature were maintained at 500 µL/min and 40 °C. Aliquots of 10 μL of the extracted samples were injected. For quantitative analysis, the optimized selective reaction monitoring (SRM) mode with the following parameters: *m*/*z* 138.1 > 123.0 (product ion mass used for the quantification of analyte), 138.1 *m*/*z* → 94.1 *m*/*z* was used.

### 4.11. Effect of EMHPS on the Expression of ABCB1 and SLCO1B1

To test the effect of EMHPS on the expression of ABCB1 and SLCO1B1, Caco-2 or HepG2 cells, respectively, were cultured on a 6-well plate. EMHPS was used at concentrations of 1, 10, and 100 µM, and the duration of exposure was 24 h. After the incubation, the cells were removed from the surface of the wells with a trypsin–EDTA solution (0.25% trypsin and 0.2% EDTA, Sigma-Aldrich, St. Louis, MO, USA).

The expression levels of ABCB1 and SLCO1B1 were analyzed via Western blot.

### 4.12. Western Blot

Cells were lysed with NP40 Cell Lysis Buffer Thermo (Thermo Fisher Scientific) with protease inhibitors (Sigma Aldrich, St. Louis, MO, USA) for 30 min at +4 °C with constant stirring at a rate of 10^7^ cells per 100 µL of buffer. Protein concentrations were quantified with the Pierce Coomassie Plus Assay Kit (Thermo Fisher, Rockford, IL, USA). Proteins (20 μg/sample) were separated using 10% SDS–PAGE and transferred onto nitrocellulose membranes using a semidry blotting system (Transblot, Bio-Rad, California, USA). Subsequently, the membranes were blocked for 1 h with TBS 1% Casein Blocker (Bio-Rad) and then incubated overnight at 4 °C with primary antibodies (P-Glycoprotein Antibody MA5-13854, Invitrogen; OATP2 Polyclonal Antibody, PA5-113548, Invitrogen). The membranes were washed in TBS and then incubated with secondary antibodies (Rabbit-anti-Mouse IgG (H + L), HRP, Invitrogen for ABCB1 and Goat anti-Rabbit IgG (H + L) Cross-Adsorbed Secondary Antibody, HRP, Invitrogen for SLCO1B1) for 1 h at room temperature.

The protein content was measured relative to the level of housekeeping protein GAPDH (primary antibodies: GAPDH Loading Control Monoclonal Antibody (GA1R) and DyLight 68 (Invitrogen); secondary antibodies: secondary rabbit antibodies to primary GAPDH antibodies—Rabbit-anti-Mouse IgG (H + L) Secondary Antibody and HRP (Invitrogen). Chemiluminescence was recorded using ChemiDocXRS+ (Bio-Rad). The intensity of the obtained bands was analyzed densitometrically using ImageLab 6.0.0 software (Bio-Rad).

### 4.13. Data Analysis

Statistical analysis was conducted using GraphPad Prism version 8.1.2. The data are presented as mean ± standard deviation (SD). Differences among groups were determined using ANOVA, followed by Tukey’s test and unpaired *t*-tests. *p*-values of <0.05 were considered to be statistically significant.

For inhibition studies, fexofenadine ER in Caco-2 cells and atorvastatin uptake in HEK293-SLCO1B1 cells were converted to the percentage of vehicle control.

Half maximal inhibitory concentration (IC_50_) values were determined through nonlinear regression based on the three-parameter logistic function.

## 5. Conclusions

Thus, the study has found that EMHPS is not a substrate of ABCB1 and SLCO1B1, but that it inhibits the activities of these transporters in vitro. SLCO1B1 inhibition and systemic ABCB1 inhibition are not clinically relevant, but ABCB1 inhibition by EMHPS in the gastrointestinal tract needs to be tested in vivo through clinical trials.

## Figures and Tables

**Table 1:** Transport of EMHPS across the Caco-2 cells monolayer (M ± SD, cm/s).

Parameter	10 µM	100 µM	250 µM
Papp b-a	7.56 × 10^−6^ ± 2.98 × 10^−6^	6.63 × 10^−6^ ± 2.15 × 10^−6^	1.65 × 10^−5^ ± 4.16 × 10^−6^
Papp a-b	7.66 × 10^−6^ ± 1.71 × 10^−6^	7.05 × 10^−6^ ± 2.15 × 10^−6^	2.23 × 10^−5^ ± 1.99 × 10^−6^
ER	0.99 ± 0.323	0.94 ± 0.039	0.75 ± 0.26

**Table 2:** The effect of EMHPS on the transport of fexofenadine through the Caco-2 cells monolayer, * *p* < 0.05, ** *p* < 0.01, *** *p* < 0.01—differences with control, ANOVA, post hoc Tukey’s test.

Concentration of EMHPS	Papp b-a, cm/s	Papp a-b, cm/s	ER
Control	3.64 × 10^−6^ ± 0.21 × 10^−6^	0.61 × 10^−6^ ± 0.21 × 10^−6^	6.39 ± 2.04
1 µM	4.2 × 10^−6^ ± 0.3 × 10^−6^	1.1 × 10^−6^ ± 0.27 × 10^−6^	3.94 ± 0.75
10 µM	4.15 × 10^−6^ ± 0.67 × 10^−6^	1.22 × 10^−6^ ± 0.29 × 10^−6^	3.64 ± 1.50
50 µM	3.61 × 10^−6^ ± 1.19 × 10^−6^	1.29 × 10^−6^ ± 0.87 × 10^−6^	3.18 ± 0.89 *
100 µM	2.57 × 10^−6^ ± 0.27 × 10^−6^	1.54 × 10^−6^ ± 0.4 × 10^−6^	1.73 ± 0.37 **
200 µM	1.73 × 10^−6^ ± 0.18 × 10^−6^ *	2.9 × 10^−6^ ± 0.29 × 10^−6^*	0.6 ± 0.12 ***

**Table 3:** The effect of succinic acid on the transport of fexofenadine through the Caco-2 cells monolayer.

Concentration of EMHPS	Papp b-a, cm/s	Papp a-b, cm/s	ER
Control	3.64 × 10^−6^ ± 0.21 × 10^−6^	0.61 × 10^−6^ ± 0.21⋅10^−6^	6.39 ± 2.04
1 µM	3.45 × 10^−6^ ± 0.43 × 10^−6^	0.65 × 10^−6^ ± 0.13 × 10^−6^	5.55 ± 1.86
10 µM	3.75 × 10^−6^ ± 0.19 × 10^−6^	0.66 × 10^−6^ ± 0.12 × 10^−6^	5.79 ± 0.97
50 µM	4.06 × 10^−6^ ± 0.21 × 10^−6^	0.73 × 10^−6^ ± 0.04 × 10^−6^	5.5 ± 0.35
100 µM	3.39 × 10^−6^ ± 0.38 × 10^−6^	0.52 × 10^−6^ ± 0.11 × 10^−6^	6.69 ± 1.53
200 µM	3.98 × 10^−6^ ± 0.65 × 10^−6^	0.64 × 10^−6^ ± 0.11 × 10^−6^	6.41 ± 1.89

**Table 4:** The effect of verapamil on the transport of fexofenadine through the Caco-2 cells monolayer, * *p* < 0.05, ** *p* < 0.01, *** *p* < 0.01—differences with control, ANOVA, post hoc Tukey’s test.

Concentration of Verapamil	Papp b-a, cm/s	Papp a-b, cm/s	ER
Control	3.64 × 10^−6^ ± 0.21 × 10^−6^	0.61 × 10^−6^ ± 0.21 × 10^−6^	6.39 ± 2.04
1 µM	3.89 × 10^−6^ ± 0.80 × 10^−6^	0.76 × 10^−6^ ± 0.26 × 10^−6^	5.29 ± 0.77
10 µM	3.2 × 10^−6^ ± 0.5 × 10^−6^	0.85 × 10^−6^ ± 0.31 × 10^−6^	4.01 ± 1.56
50 µM	1.47 × 10^−6^ ± 0.25 × 10^−6^ ***	0.55 × 10^−6^ ± 0.2 × 10^−6^	2.81 ± 0.69 *
100 µM	1.15 × 10^−6^ ± 0.4 × 10^−6^ ***	0.67 × 10^−6^ ± 0.15 × 10^−6^	1.82 ± 0.98 **
200 µM	1.23 × 10^−6^ ± 0.19 × 10^−6^ ***	1.46 × 10^−6^ ± 0.43 × 10^−6^ *	0.89 ± 0.26 **

## Data Availability

Data is contained within the article.
